# Associations between sensory processing difficulties and dysregulation/deficient self-regulation in preschool children with autism spectrum disorder

**DOI:** 10.3389/fpsyg.2026.1768987

**Published:** 2026-07-08

**Authors:** Yao Lee, Yi-Shan Sung, Shan-Chu Yang, Ling-Yi Lin

**Affiliations:** 1 Department of Occupational Therapy, College of Medicine, National Cheng Kung University, Tainan City, Taiwan; 2 Department of Occupational Therapy, Asia University, Taichung City, Taiwan; 3 Good Doctor Clinic, Kaohsiung City, Taiwan; 4 Institute of Allied Health Sciences, College of Medicine, National Cheng Kung University, Tainan City, Taiwan

**Keywords:** autism spectrum disorder, emotional dysregulation, preschool children, sensory processing, short sensory profile-2

## Abstract

**Background:**

Sensory processing difficulties and dysregulation/deficient self-regulation are prevalent features in preschool-aged children with autism spectrum disorder (ASD), yet the specific associations between distinct sensory patterns and dysregulation/deficient self-regulation remain underexplored, particularly in non-Western populations.

**Objective:**

This study aimed to investigate the associations between sensory processing patterns, measured by the Short Sensory Profile–2 (SSP–2), and dysregulation/deficient self-regulation, indexed by the Child Behavior Checklist–Dysregulation Profile (CBCL–AAA), in a cohort of Taiwanese preschoolers with ASD.

**Methods:**

A total of 80 preschool children with ASD (aged 36–73 months) were recruited for this cross-sectional study. Sensory processing patterns were categorized into Seeker, Avoider, Sensor, and Bystander quadrants using the SSP-2. Dysregulation/deficient self-regulation was quantified using the CBCL–AAA composite score, which aggregates Attention Problems, Aggressive Behavior, and Anxious/Depressed subscales.

**Results:**

The results indicated a high prevalence of atypical sensory processing, with most participants scoring in the “more/much more than others” range for the Avoider and Sensor patterns. Pearson’s correlation analysis revealed significant positive associations between sensory processing difficulties and dysregulation/deficient self-regulation. Specifically, the Seeker, Avoider, and Sensor quadrants demonstrated strong correlations with the CBCL–AAA score, whereas the Bystander quadrant showed a moderate correlation. Both Sensory and Behavior composite scores were strongly associated with the severity of dysregulation/deficient self-regulation.

**Conclusion:**

These findings suggest that heightened sensory reactivity, particularly in low-threshold patterns (Avoider and Sensor) as well as sensory seeking behaviors, is closely linked to dysregulation/deficient self-regulation in young children with ASD. The study highlights the clinical importance of profiling sensory patterns to identify children at elevated risk for dysregulation. Interventions that directly target sensory processing strategies may provide a critical pathway for supporting self-regulation in preschool children with ASD.

## 1 Introduction

Autism Spectrum Disorder (ASD) is a neurodevelopmental disorder characterized by impairments in social interaction and communication, and repetitive patterns of behavior, interests, and activities (American Psychiatric Association, 2022). In the Taiwan national epidemiological study of child mental disorders, the estimated lifetime prevalence of ASD was approximately 1.0% (Chen et al., 2020). Using the national disability registry of Taiwan (ages 3–17, from 2004 to 2010), the male-to-female prevalence ratio for ASD was approximately 5.6–6.1 to 1 (Lai et al., 2012). The Diagnostic and Statistical Manual of Mental Disorders–Fifth Edition (DSM–5) explicitly includes sensory symptoms as part of the diagnostic criteria for ASD (American Psychiatric Association, 2022). Previous studies have reported that up to 90% of people with ASD have sensory processing difficulties (Marco et al., 2011). These symptoms may result in problems participating in daily life and social activities across the lifespan. Sensory processing difficulties and emotional dysregulation are both highly prevalent and impairing in preschool children with ASD (Gigliotti et al., 2025). Yet, their specific associations, particularly by distinct sensory patterns, remain under-characterized, especially in non-Western contexts.

According to Dunn’s sensory processing framework (Dunn, 1997), individual differences in the way people detect, modulate, and respond to stimuli are based on two key dimensions: neurological threshold (high and low) and self-regulation strategy (active and passive). This model yields four distinct sensory processing patterns: low registration (high threshold, passive response), sensation seeking (high threshold, active response), sensory sensitivity (low threshold, passive response), and sensation avoiding (low threshold, active response; Dunn, 2001).

Empirical studies using Dunn’s framework and standardized tools such as the Short Sensory Profile and Short Sensory Profile-2. The typical sensory profile score for preschool-aged children with autism spectrum disorder is significantly lower than that of typically developing peers, indicating marked sensory processing dysfunction across multiple domains. On standardized measures such as the Short Sensory Profile (SSP) and Short Sensory Profile–2 (SSP–2), 95% of preschool children with ASD score in the “definite difference” or “probable difference” range, reflecting substantial difficulties in areas including tactile sensitivity, auditory filtering, and under-responsiveness/seeking sensation (Tomchek and Dunn, 2007; Lin, 2020). These sensory processing patterns are associated with challenges in social participation, adaptive behavior, and engagement in daily activities (Lin, 2020; Tomchek et al., 2015).

The emotional dysregulation problem in children with ASD is characterized by persistent difficulties in monitoring, modulating, and expressing emotions, often manifesting as intense emotional reactions, irritability, tantrums, mood fluctuations, and reactive aggression (Davico et al., 2022; Northrup et al., 2024; Lin, 2025). These children frequently employed fewer adaptive emotion regulation strategies, such as problem-solving or cognitive reappraisal. They relied more on maladaptive strategies, including avoidance, repetitive behaviors, or suppression, which exacerbated their emotional and behavioral challenges (Samson et al., 2015; Restoy et al., 2024).

Emotional dysregulation in ASD was associated with impaired social functioning, increased risk of internalizing and externalizing symptoms, and significant functional impairment in daily life and adaptive skills (Davico et al., 2022; Northrup et al., 2024). The severity of emotional dysregulation correlated with core autistic traits, sleep problems, and parental mental health, and is often persistent from early childhood into adolescence, especially in the context of family stress or parental depression (Northrup et al., 2024; Bennett et al., 2025).

A growing body of research shows that atypical sensory processing is consistently linked to emotional dysregulation or behavioral problems in children with ASD (Chen et al., 2024; Brandes-Aitken et al., 2024). Sensory hyperresponsiveness, hypo-responsiveness, and sensory seeking have all been linked to both internalizing symptoms and externalizing symptoms (Chen et al., 2024; Brandes-Aitken et al., 2024). Emotional dysregulation appears to serve as a key mediator between sensory processing difficulties and behavior problems, such that greater sensory processing difficulties predict higher levels of maladaptive behaviors via increased dysregulation, including irritability, tantrums, and social withdrawal (Sung et al., 2024). Distinct sensory processing subtypes are associated with specific behavioral and emotional profiles. For example, sensory over-responsivity is most strongly related to anxiety and emotion dysregulation, whereas sensory seeking and under-responsiveness are more closely tied to attention deficit hyperactivity disorder-like behaviors and externalizing problems (Chen et al., 2024; Brandes-Aitken et al., 2024). Longitudinal findings indicate that persistent sensory hyperresponsiveness and hypo-responsiveness in early childhood predict poorer adaptive functioning and greater maladaptive behaviors in later childhood (Chen et al., 2025).

The Child Behavior Checklist–Dysregulation Profile (CBCL–DP), operationalized as the composite of the Attention Problems, Aggressive Behavior, and Anxious/Depressed subscales (often referred to as CBCL-AAA), provides a validated dimensional index of broad dysregulation and deficient self-regulation in children with ASD (Keefer et al., 2020). This composite is grounded in robust empirical evidence demonstrating that these three syndromes covary meaningfully to form a superordinate factor representing a severe deficit in self-regulation (Keefer et al., 2020; Jucksch et al., 2011). Rather than capturing “emotion” alone, this composite reflects co-occurring affective, behavioral, and cognitive regulation difficulties and has therefore been conceptualized in the literature as a measure of dysregulation or deficient emotional self-regulation (DESR). Although attention is inherently a cognitive construct rather than an emotion, contemporary models of emotion regulation clarify its indispensability in regulatory processes. According to Gross’s (1998) Process Model of Emotion Regulation, attentional deployment (e.g., distraction, concentration, and shifting attention away from distressing stimuli) serves as a primary cognitive mechanism through which individuals modulate their emotional arousal (Gross, 1998). Consequently, attention problems reflect a deficit in the executive, top-down cognitive processes required to inhibit impulsive emotional responses. Impaired attentional capacity therefore acts as a fundamental neurocognitive contributor of dysregulation.

Extensive empirical evidence has validated the CBCL–DP/AAA as a robust clinical proxy for dysregulation, with strong psychometric properties and predictive validity (Keefer et al., 2020; Holtmann et al., 2011). By simultaneously capturing the behavioral, cognitive, and affective dimensions of regulation, this profile has been shown to be highly predictive of severe mood dysregulation, pediatric bipolar disorder, ASD, and profound emotional lability (Biederman et al., 1995; Holtmann et al., 2011). Elevated CBCL–AAA scores reflect broad difficulties regulating affect, behavior, and cognition, and have been strongly associated with the presence and severity of emotional dysregulation in ASD populations (Davico et al., 2022; Keefer et al., 2020; Joshi et al., 2018; Geeraerts et al., 2015). In autistic children, higher CBCL–AAA scores are linked to increased irritability, mood lability, tantrums, and reactive aggression, as well as poorer adaptive functioning and greater psychosocial impairment (Davico et al., 2022; Joshi et al., 2018). Because the AAA composite captures both internalizing (anxiety/depression) and externalizing (aggression, attention problems) facets of dysregulation and is predictive of more severe clinical morbidity and functional impairment, it is widely used in research and clinical practice to identify and quantify dysregulation in ASD (Keefer et al., 2020; Joshi et al., 2018; Geeraerts et al., 2015). In the present study, we therefore use the CBCL–AAA composite as an index of broad dysregulation/deficient self-regulation, with a particular focus on its relevance for emotional and behavioral regulation in preschool children with ASD. However, we acknowledge that this profile is broader than emotion alone and also captures attention and behavioral regulation difficulties.

Despite evidence connecting atypical sensory processing with behavioral problems in ASD, several gaps remain. First, many studies rely on broad-band or syndrome scales rather than specifically using the CBCL–AAA composite to index broad dysregulation/deficient self-regulation, including prominent emotional dysregulation. Second, few studies focus on preschool autistic children or examine pattern-specific associations (Seeker, Avoider, Sensor, Bystander; Sensory/Behavior composites) between detailed sensory processing profiles and dysregulation. Third, Chinese-speaking samples are underrepresented, limiting the generalizability of current findings. Addressing these gaps, the present study examines whether specific SSP–2 sensory patterns and composites (e.g., Seeker, Avoider, Sensor, Bystander; sensory/behavior composites) are positively associated with CBCL–AAA scores in preschool children with ASD. Clarifying these pattern-specific associations is theoretically informative, clinically actionable (supporting early risk identification, AAA-based stratification, and selection of sensory-informed intervention targets), and methodologically valuable by providing robust, context-specific effect sizes to inform future mediation and intervention research. Guided by this rationale, the study asks the following questions: (a) What are the rates of co-occurring dysregulation/deficient self-regulation in preschool children with ASD who manifested sensory processing difficulties?; and (b) Are SSP–2 quadrants and composite scores positively associated with CBCL–AAA?

## 2 Materials and methods

### 2.1 Participants

This was a cross-sectional, correlational study examining associations between sensory processing and dysregulation/deficient self-regulation in preschool children with ASD. A total of 80 preschool children with ASD were recruited using a convenience sampling method from clinics, hospitals and early intervention centers from November 2023 to December 2024. The inclusion criteria were as follows: (a) children aged 36 to 83 months; (b) children with a diagnosis of ASD according to the Diagnostic and Statistics Manual of Mental Disorders – Fifth edition (DSM–5) by registered pediatric psychiatrists; (c) mild to severe symptoms of ASD according to the Standard Version of Childhood Autism Rating Scale – Second edition (CARS–2); and (c) the presence of no visual or hearing impairment. Initially, we approached 82 potential participants. After applying the inclusion criteria, the number of eligible participants approached were 80. Two participants (2.4%) who had a CARS-2 scores below 30 were excluded.

### 2.2 Measurements

#### 2.2.1 Childhood autism rating scale—second edition

Clinicians completed the CARS–2, a clinician-rated instrument for identifying autism characteristics and grading severity based on direct observation. CARS–2 comprises 15 items scored from 1 to 4 in 0.5-point increments. Summed total scores (range 15–60) classify severity as likely nonautistic (15–29.5), Mild to moderate level of behaviors related to autism (30–36.5), or severe level of behaviors related to autism (37–60). The internal consistency estimate of 0.93 was obtained. The sensitivity value was 0.88 and the specificity value was 0.86. The positive predictive value was 0.88 and negative predictive value was 0.87 (Schopler et al., 2010).

#### 2.2.2 Chinese version of Peabody picture vocabulary test—revised

The PPVT–R assesses the receptive vocabulary of children aged 3 to 12 (Lu and Liu, 1994). Children heard a spoken word and indicated (by pointing or saying the number) which of four pictures best matched the word. The PPVT–R includes two parallel forms (L and M), each with 175 items, and uses standard basal/ceiling rules to determine starting and stopping points; raw scores are converted to age equivalents and standard scores (Bracken et al., 1984). Significant correlations were found between the Wechsler Intelligence Scale for Children–Revised (WISC–R) Full Scale intelligence quotient (IQ; 0.46) and the WISC–R Verbal IQ (0.50) and the PPVT–R (Beck and Black, 1986).

#### 2.2.3 Short sensory profile—second edition

SSP–2 was a parent reported questionnaire measuring sensory processing patterns in 3 years old to 14 years and 11 months old children. SSP–2 contained 34 items derived from Child Sensory Profile 2, which was a five-point Likert scale, ranging from 1 (almost never) to 5 (almost always). There were 6 scores available on SSP–2. The first four scores represent the sensory patterns from Dunn’s Sensory Processing Framework (i.e., Seeking, Avoiding, Sensitivity, Registration). There were also summary scores for sensory items collectively and a behavior summary score. The Sensory composite aggregates modality-specific sensory responses (e.g., auditory, visual, touch, movement, oral), whereas the Behavior composite summarizes everyday behavioral/emotional responses that may be influenced by sensory processing (e.g., distractibility, avoidance, arousal/soothing). Because the Behavior composite includes regulatory and behavioral content, it may conceptually overlap with components of the CBCL–AAA profile, particularly Attention Problems and Aggressive Behavior. Therefore, in the present study, the four SSP–2 quadrant scores were treated as the primary theoretically grounded indicators of sensory processing patterns, whereas the Sensory and Behavior composites were interpreted as broader supplementary indicators. The SSP–2 has good internal consistency (Cronbach’s *α* range of 0.79– 0.86) and convergent validity with the behavior assessment system for children–second edition (the correlation range of *r* = −0.56 to −0.78, *p* < 0.01; Dunn, 2014).

#### 2.2.4 Child behavior checklist

Caregivers completed the Traditional Chinese version of the CBCL/1.5–5, a parent-report inventory of 99 problem items rated 0–2 (0 = Not True, 1 = Somewhat/Sometimes True, 2 = Vey True/Often True; Achenbach and Rescorla, 2000; Chen et al., 2009). Raw scores are converted to age- and sex-normed T-scores. CBCL yields seven syndrome scales (Emotionally Reactive, Anxious/Depressed, Somatic Complaints, Withdrawn, Sleep Problems, Attention Problems, Aggressive Behavior), broad-band scales (Internalizing, Externalizing, Total Problems), and DSM-oriented scales based on international expert mappings to DSM criteria. For the CBCL/1.5–5, the test–retest reliability is approximately 0.85, and the stability coefficient of the scale scores is about 0.61, indicating good reliability and satisfactory stability. In Taiwan, studies have likewise reported acceptable reliability and validity for the CBCL/1.5–5 the test–retest reliability = 0.52–0.84; construct validity = 0.37–0.91 (Wu et al., 2012). In this study, the T-scores from the Attention Problems, Aggressive Behavior, and Anxious/Depressed subscales are combined to create the CBCL–AAA composite score, corresponding to the well-established CBCL Dysregulation Profile (CBCL–DP). Consistent with prior work, this composite was used to index broad dysregulation/deficient self-regulation, including prominent emotional dysregulation, and a total score of 180 or higher was taken as the cutoff for a clinically elevated dysregulation profile.

### 2.3 Procedures

The study protocol has been approved by the Institutional Review Board (A-BR-112-055) at National Cheng Kung University Hospital. Initially, the case manager obtained written informed consent and interviewed caregivers for their concerns and demographic characteristics. In the beginning, the investigator used CARS–2 and PPVT–R to screen participants’ autism severity and cognitive function. Next, in order to gather information about children’s sensory processing patterns and emotional dysregulation, the caregivers needed to fill out the SSP–2 and CBCL, respectively.

### 2.4 Data analysis

Descriptive statistics (such as frequencies, means, and standard deviations) were analyzed using SPSS 29.0. Pearson correlation coefficients were used to examine the relationships between sensory processing patterns and dysregulation/deficient self-regulation among these participants, with the significance level set at *p* < 0.008 following Bonferroni correction for multiple correlations. We categorized the magnitude of the Pearson correlation coefficients as weak (*r* ≤ 0.39), moderate (0.4–0.69), and strong (*r* ≥ 0.7; Schober et al., 2018). In addition, we conducted a post-hoc power analysis for the significant correlations. Given our sample size (*n* = 80), the observed effect sizes (*r* = 0.60–0.75), and the Bonferroni-adjusted alpha level (*p* < 0.008), the estimated statistical power was approximately 0.96, indicating adequate power to detect the observed associations.

## 3 Results

Table 1 presents the sample characteristics, along with mean scores for sensory processing patterns on the SSP–2 and dysregulation/deficient self-regulation on the CBCL–AAA (indexing broad dysregulation/deficient self-regulation, including prominent emotional dysregulation). The majority of participants were male (83.8%; *n* = 67), while females comprising 16.2% (*n* = 13). The mean score of PPVT–R was 99.5 (SD = 20.6), with 20 children scoring below 85. The mean total score of CARS–2 was 33.9 (SD = 4.3; range = 30–49), reflecting mild-to-moderate to severe ASD symptom severity. SSP–2 quadrant means indicated atypical sensory processing patterns: Sensor (M = 30.0, SD = 7.3; range = 16–50), Avoider (M = 29.0, SD = 7.2; range = 13–42), Bystander (M = 21.3, SD = 7.1; range = 0–35), and Seeker (M = 18.4, SD = 6.2; range = 8–33). Section composites showed Sensory (M = 36.9, SD = 11.4; range = 16–62) and Behavior (M = 61.2, SD = 14.2; range = 28–85). For dysregulation/deficient self-regulation, the CBCL–AAA mean score was 188.8 (SD = 25.3; range = 150–238); 40.0% (*n* = 32) scored below 180 (no dysregulation profile), while the 60.0% (*n* = 48) fell in the dysregulation range. Given the small, clinic-based nature of this sample, these proportions should be interpreted as descriptive of this cohort rather than as population-level prevalence estimates.

**Table 1:** Characteristics and scores in sensory processing patterns and dysregulation/deficient self-regulation of participants with ASD.

Variable	Mean (SD)/*n* (%)	Range
Age (month)	56.1 (10.8)	36–73
Gender		
Male	67 (83.8%)	
Female	13 (16.2%)	
PPVT–R score	99.5 (20.6)	49–145
Score < 85	20 (25.0%)	
CARS–2 Score	33.9 (4.3)	30–49
SSP–2		
Seeker	18.4 (6.2)	8–33
Avoider	29.0 (7.2)	13–42
Sensor	30.0 (7.3)	16–50
Bystander	21.3 (7.1)	0–35
Sensory composite	36.9 (11.4)	16–62
Behavior composite	61.2 (14.2)	28–85
CBCL–AAA Score	188.8 (25.3)	150–238
Score < 180	32 (40.0%)	

ASD: autism spectrum disorder; PPVT–R: Peabody picture vocabulary test–revised; CARS-2: childhood autism rating scale-second edition; CBCL: child behavior checklist; SSP–2: short sensory profile–2.

Table 2 shows frequency distributions of SSP–2 scores, revealing a high prevalence of atypical sensory processing, particularly in Avoider, Bystander, and Behavior sections, with over half categorized as “much more than others.” Percentages scoring more than most people across four quadrants were: Avoider (81.2%, *n* = 65), Sensor (78.7%, *n* = 63), Bystander (72.6%, *n* = 58), and Seeker (50.0%, *n* = 40). Avoider and Sensor patterns were most common, followed by Bystander and Seeker; few scored in typical (“just like the majority”) or under-responsive (“much less than others”) ranges, highlighting pervasive processing difficulties in this preschool ASD sample.

**Table 2:** Frequency of SSP–2 sensory processing scores.

SSP–2	Much less than others (*N*)/*n* (%)	More less than others (*N*)/*n* (%)	Just like the majority (*N*)/*n* (%)	More than others (*N*)/*n* (%)	Much more than others (*N*)/*n* (%)
Seeker	0 (0%)	0 (0%)	40 (50.0%)	20 (25.0%)	20 (25.0%)
Avoider	0 (0%)	0 (0%)	15 (18.8%)	25 (31.2%)	40 (50.0%)
Sensor	0 (0%)	0 (0%)	17 (21.3%)	29 (36.2%)	34 (42.5%)
Bystander	1 (1.1%)	0 (0%)	21 (26.3%)	13 (16.3%)	45 (56.3%)
Sensory composite	0 (0%)	0 (0%)	29 (36.2%)	19 (23.8%)	32 (40.0%)
Behavior composite	0 (0%)	0 (0%)	12 (15.0%)	23 (28.7%)	45 (56.3%)

SSP–2: short sensory profile 2.

**Figure 1:**
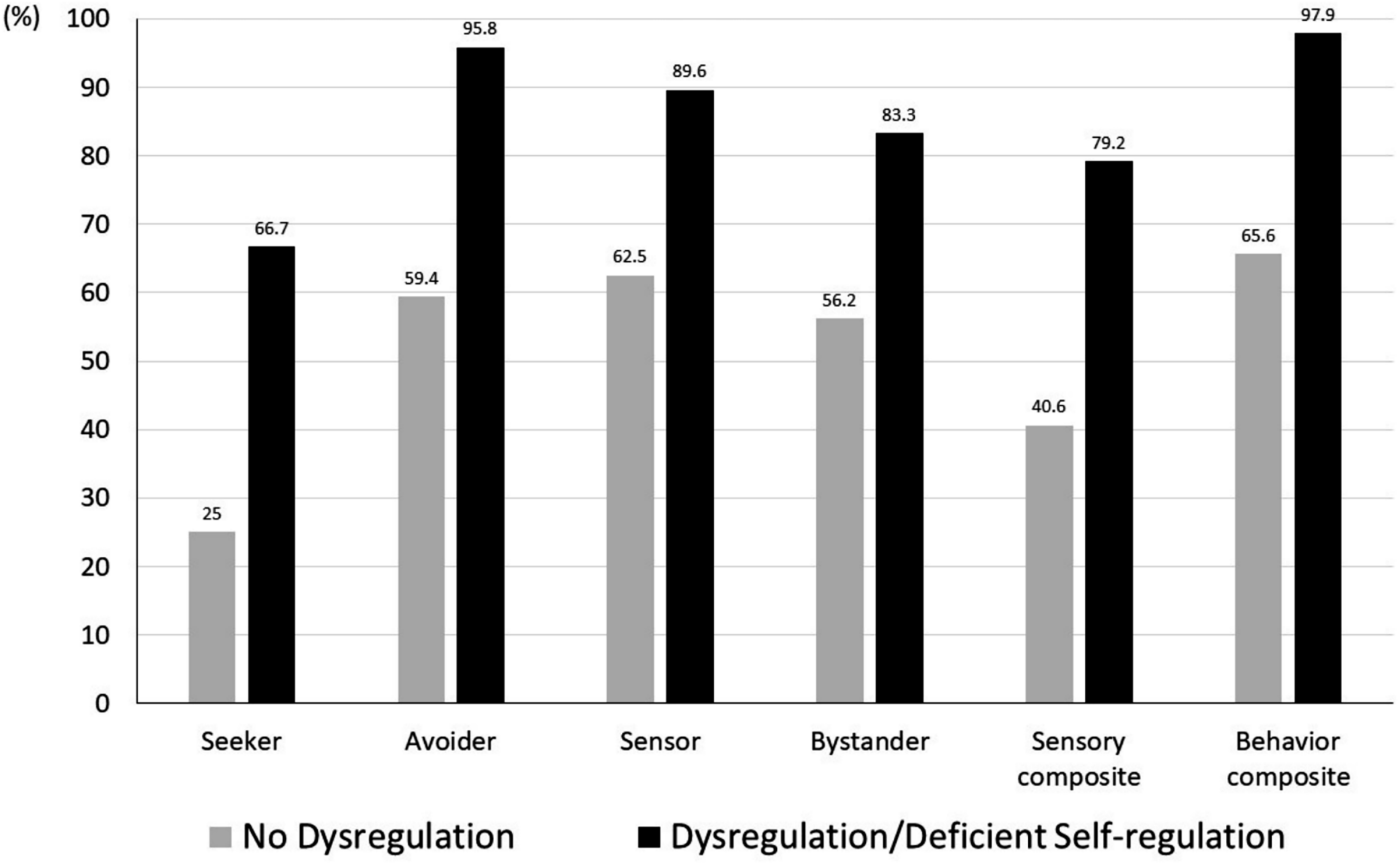
The rates of co-occurring dysregulation/deficient self-regulation in preschool children with ASD who manifested sensory processing difficulties. Percentages scoring more/much more than most people across four quadrants and two section composites.

Data from Figure 1 indicate that sensory processing difficulties are highly prevalent in children with ASD and dysregulation/deficient self-regulation (66.7–97.9%). Specifically, regarding the Behavior composite and Avoider patterns, nearly all children with dysregulation/deficient self-regulation (over 95%) exhibited significant sensory processing difficulties.

Pearson’s correlations (Table 3) demonstrated significant associations between SSP-2 subscales/composites and CBCL–AAA scores. Quadrant correlations were strongest for Avoider (*r* = 0.741, *p* < 0.001), followed by Seeker (*r* = 0.636, *p* < 0.001) and Sensor (*r* = 0.639, *p* < 0.001), with Bystander showing moderate association (*r* = 0.464, *p* < 0.001). Composites likewise correlated robustly: Sensory (*r* = 0.613, *p* < 0.001) and Behavior (*r* = 0.735, *p* < 0.001). Partial correlation analysis was employed to detect possible effects of autism severity on the relationship between SSP-2 subscales/composites and CBCL–AAA scores. The correlations between the SSP-2 subscales/composites and CBCL–AAA scores remained significant after partialling the effects of autism severity (Table 3). Additionally, partial correlation analysis was employed to detect possible effects of the receptive vocabulary on the relationship between SSP-2 subscales/composites and CBCL–AAA scores. The correlations between the SSP-2 subscales/composites and CBCL–AAA scores remained significant after partialling the effects of receptive vocabulary (rs = 0.351–0.913, *p* < 0.008). These results confirm that greater sensory processing difficulties across patterns and domains are consistently linked to higher dysregulation/deficient self-regulation severity.

**Table 3:** Correlation between SSP-2 and CBCL-AAA score.

Variables	1	2	3	4	5	6	7	8
1 SSP–2-seeker	–							
2 SSP–2-avoider	0.605^***^ (0.605^***^)	–						
3 SSP–2-sensor	0.756^***^ (0.756^***^)	0.720^***^ (0.720^***^)	–					
4 SSP–2-bystander	0.515^***^ (0.518^***^)	0.506^***^ (0.512^***^)	0.570^***^ (0.578^***^)	–				
5 SSP–2-sensory	0.866^***^ (0.866^***^)	0.630^***^ (0.631^***^)	0.847^***^ (0.847^***^)	0.663^***^ (0.666^***^)	–			
6 SSP–2-behavior	0.686^***^ (0.687^***^)	0.914^***^ (0.914^***^)	0.830^***^ (0.829^***^)	0.593^***^ (0.602^***^)	0.696^***^ (0.697^**^)	–		
7 CBCL–AAA score	0.636^***^ (0.637^***^)	0.741^***^ (0.743^***^)	0.639^***^ (0.641^***^)	0.464^***^ (0.463^***^)	0.613^***^ (0.613^***^)	0.735^***^ (0.739^***^)	–	
8 CBCL–anxiety/depression	0.401^***^ (0.401^***^)	0.552^***^ (0.551^***^)	0.520^***^ (0.519^***^)	0.308^**^ (0.312^**^)	0.467^***^ (0.467^***^)	0.528^***^ (0.528^***^)	0.699^***^ (0.700^***^)	–

**p < 0.008, ***p < 0.001; SSP–2: short sensory profile 2; CBCL: child behavior checklist. Partial correlation controlling for autism severity (in parentheses).

## 4 Discussion

This study examined associations between sensory processing difficulties and dysregulation/deficient self-regulation in preschool children with ASD. Results revealed that over 60% of children with ASD who co-occur with dysregulation/deficient self-regulation manifested sensory processing difficulties. Robust positive correlations suggest that greater sensory processing differences are associated with a heightened severity of dysregulation/deficient self-regulation in early childhood.

In this preschool ASD cohort, SSP–2 score distributions were right-shifted and more extreme than in prior reports. Compared with a Taiwanese preschool sample (Lin, 2020), quadrant means exceeded typical-range cutoffs, with low-threshold quadrants (Sensor, Avoider) especially prominent extending patterns observed by Lin (2020) but to a greater degree here. Categorical analyses showed large proportions in “more/much more than others” bands, particularly for Avoider, Sensor, and Bystander; composites likewise reflected marked atypicality in Sensory and Behavior domains. This profile suggests that everyday stimuli readily provoke over-reactivity and regulatory demands consistent with the elevated CBCL–AAA scores observed in this sample.

These distributions align with earlier large-sample work using the original SSP by Tomchek and Dunn (2007), which documented pervasive impairments in autistic children, including about 95% with total score deficits and high rates in under-responsive/seeking and auditory filtering domains (Tomchek and Dunn, 2007). The elevated Seeker and Bystander proportions in our SSP–2 data mirror these robust features.

All SSP–2 subscales and composites significantly correlated with the CBCL–AAA score. However, strong correlations were observed specifically for the Seeker, Avoider, and Sensor subscales, whereas the correlation for the Bystander subscale was moderate. The low-threshold sensory processing patterns (Sensor, Avoider) exhibited the strongest links, indicating hyper-arousal and negative emotions in response to sensory input. This heightened reactivity to stimuli has been associated with increased physiological arousal and emotional distress for children (Chen et al., 2024; Ben-Sasson et al., 2008; Green et al., 2015). Children who display sensory sensitivity (Sensor) and sensory avoidance (Avoider) tend to react strongly to sensory stimuli, which have been associated with elevated physiological responses and emotional distress in reaction to typical everyday sensory experiences (Chen et al., 2024; Ben-Sasson et al., 2008; Green et al., 2015). Furthermore, the slightly weaker correlation for the Bystander pattern may be attributed to the nature of the CBCL–AAA composite, which focuses heavily on active dysregulation, such as aggression and attention issues. The high-threshold, passive pattern is more likely to manifest as withdrawal rather than overt behavioral problems.

It is important to note that the SSP-2 Behavior composite includes items tapping distractibility, arousal regulation, avoidance, and emotional reactions, which conceptually overlap with CBCL Attention Problems and Aggressive Behavior domains. Accordingly, the strong association between the Behavior composite and CBCL-AAA should be interpreted cautiously, as it likely partly reflects shared measurement content and common method variance rather than a purely independent link between sensory processing and dysregulation. For this reason, our interpretation places greater emphasis on the sensory quadrants and the Sensory composite, which provide a more theoretically distinct index of sensory processing differences.

Neurobiological research shows that sensory over-responsivity is linked to increased activity in limbic regions and primary sensory cortices in individuals with ASD. This connection results in heightened responses to perceived threats and challenges in regulating emotions (Green et al., 2015). Clinically, this hyper-arousal is characterized by irritability, anxiety, and a quick escalation of negative emotions when faced with aversive or unpredictable sensory input. Behavioral studies further indicate that children with heightened sensory sensitivity and avoidance tend to experience higher levels of negative emotionality, anxiety, and mood instability, regardless of the severity of their autism symptoms (Ben-Sasson et al., 2008; Narvekar et al., 2024). In our cohort, we found that this low-threshold sensory responsiveness is closely associated with dysregulation/deficient self-regulation in preschool ASD.

According to Dunn’s sensory processing theory, the Seeker sensory processing type is characterized by a high neurological threshold and active self-regulation. This means that children with this type require more intense sensory input and actively seek out sensory experiences (e.g., excessive movement, touching, or vocalizations). In contrast, the Bystander type, also referred to as Low Registration, has a high neurological threshold but utilizes passive self-regulation. As a result, these children often miss sensory cues, appear inattentive, and do not actively seek out sensory input (Dunn, 2001; Heffler et al., 2024). In children with ASD, Seeker types are associated with increased externalizing behaviors, such as hyperactivity and impulsivity, and they are prone to dysregulation/deficient self-regulation, which can manifest as difficulty modulating excitement or frustration. These children may display disruptive behaviors when their sensory needs are unmet or they become overstimulated (Chen et al., 2024; Brandes-Aitken et al., 2024; Griffin et al., 2022). On the other hand, Bystander types are more likely to exhibit withdrawn or inattentive behaviors. Their dysregulation often presents as low reactivity, poor adaptation, and difficulty engaging with the environment, which is associated with social withdrawal and negative mood (Brock et al., 2012; Weber et al., 2025). Both sensory processing types are linked to dysregulation/deficient self-regulation, but Seeker types are more strongly associated with externalizing symptoms, while Bystander types are connected to internalizing symptoms and social disengagement (Chen et al., 2024; Brock et al., 2012). Previous studies have indicated that autism and other neurodevelopmental concerns show diverse patterns of sensory processing disparities linked to higher prevalence of challenges in regulating emotions (Brandes-Aitken et al., 2024). The findings of our study align with these earlier results.

Caregiver reports and mediation analyses indicate that dysregulation/deficient self-regulation significantly mediates the relationship between sensory processing patterns and maladaptive outcomes, including sleep disturbances and increased internalizing symptoms (Sung et al., 2024; Yang and Lin, 2025). These and our findings support the clinical relevance of identifying the shared neurobiological mechanisms contributing to both sensory processing difficulties and dysregulation in children with ASD and highlight the need for integrated sensory and emotion regulation intervention. Specifically, clinical practitioners should tailor their approach to the child’s sensory profile. For children with low-threshold patterns (Sensor, Avoider) who show the strongest link to dysregulation/deficient self-regulation, interventions should prioritize environmental modifications to reduce sensory overload and anxiety. Conversely, for Seekers, providing structured sensory input may be crucial for mitigating externalizing behaviors. Identifying these specific sensory-emotional pathways in preschool years offers a critical window for targeted early intervention.

Several limitations of this study should be noted. First, cross-sectional design limits causality, we cannot determine the direction of effects. It is possible that dysregulation shapes sensory responses, that shared neurobiological mechanisms underlie both phenomena, or that shared method variance inflates associations. Longitudinal and multi-informant studies are needed to clarify these clinically plausible pathways. Second, reliance on parent reports risks shared method variance. Although the instruments are well-validated, shared method variance and parental subjective bias could inflate the observed correlations. Future studies would benefit from incorporating observational measures or teacher reports to provide a multi-informant perspective. Third, participants were recruited from hospital, clinic, and early intervention settings and the sample size was modest (*n* = 80), so the 60% rate of a CBCL-AAA dysregulation profile is not representative of the broader preschool ASD population and should be viewed as cohort-specific. Fourth, our sample had a relatively high mean PPVT–R score, representing a high-functioning cohort. Consequently, these findings may not generalize to ASD populations with co-occurring intellectual disabilities. Finally, dysregulation/deficient self-regulation was indexed indirectly via the CBCL-AAA composite, which integrates attention and behavioral dysregulation components in addition to affective symptoms. Thus, our findings should be interpreted as reflecting broad dysregulation with prominent emotional features, rather than pure emotional dysregulation.

## 5 Conclusion

This study confirms a significant association between sensory processing difficulties and dysregulation/deficient self-regulation in preschool children with ASD within a Taiwanese cohort. Specifically, our results indicate that children exhibiting Seeker, Avoider, and Sensor sensory profiles demonstrate strong correlations with dysregulation/deficient self-regulation, while the Bystander profile exhibits a moderate association. These findings support the conceptual framework that challenges in sensory processing, particularly those related to low sensory thresholds (such as Avoider and Sensor) and sensory-seeking behaviors, are associated with greater self-regulatory vulnerability in this population. Clinically, this study emphasizes the importance of incorporating sensory profiles into standard early assessments. By identifying specific sensory subtypes, clinicians and caregivers can tailor interventions to support self-regulation better and improve overall outcomes. Future longitudinal research is warranted to clarify underlying mechanisms and evaluate the long-term effectiveness of targeted sensory interventions.

## Data Availability

The raw data supporting the conclusions of this article will be made available by the authors, without undue reservation.
